# Paragangliome rétropéritonéal non secrétant: une cause rare d'occlusion intestinale haute

**DOI:** 10.11604/pamj.2014.18.312.5140

**Published:** 2014-08-20

**Authors:** Mehdi Soufi, Said Benamr, Bouziane Chad

**Affiliations:** 1 Département de Chirurgie, Faculté de Médecine d'Oujda, Université Mohammed Ier Oujda, Maroc; 2 Clinique chirurgicale B Avicenne Rabat, Rabat, Maroc

**Keywords:** Paragangliome, tumeur rétropéritonéal, chirurgie, concertation multidisciplinaire, Paraganglioma, retroperitoneal tumor, surgery, multidisciplinary concertation

## Abstract

Les paragangliomes sont des tumeurs neuroendocrines rares diagnostiquées le plus souvent chez le jeune adulte. Nous rapportons le cas rare d'une jeune femme de 20 ans présentant un paragangliome rétropéritonéal situé sur la face antérolatérale de l'aorte et comprimant l'angle duodéno-jéjunal responsable d'un syndrome subocclusif haut. Dans les formes non sécrétantes, la symptomatologie est souvent déroutante. Le diagnostic n'est généralement fait qu'après examen histologique de la pièce de résection. Le traitement de choix repose sur une chirurgie carcinologique intégrant une concertation multidisciplinaire.

## Introduction

Les paragangliomes (PG) ou phéochromocytomes extra-surrénaliens sont des tumeurs rares développées aux dépens des cellules neuro-ectodermiques du système nerveux autonome [1]. Moins de 2% se localisent en rétropéritonéal. La forme non sécrétante est exceptionnelle [2]. Elle reste longtemps asymptomatique, ce qui amène à la découvrir à des stades avancés [2]. A notre connaissance, La compression de l'angle duodéno-jéjunal est un mode de révélation jamais rapporté. Malgré les progrès de l'imagerie, cette forme pose un problème diagnostique qui n'est le plus souvent confirmé qu'après l'analyse histologique de la pièce opératoire [3]. Nous rapportons un nouveau cas dont la symptomatologie était révélée par un syndrome compressif haut chez une jeune fille de 20 ans. A travers cette observation, nous discuterons certains problèmes diagnostiques et thérapeutiques que posent ces tumeurs non sécrétantes.

## Patient et observation

**Figure 1:**
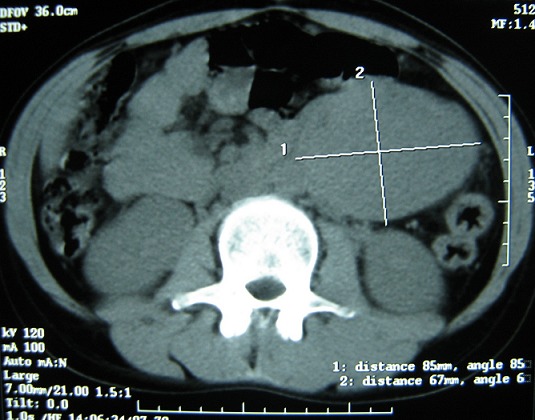
Scanner sans injection du produit de contraste montrant une tumeur occupant bien limité mesurant 9 x 7 cm prenant le contraste et ne contenant pas de calcifications

**Figure 2:**
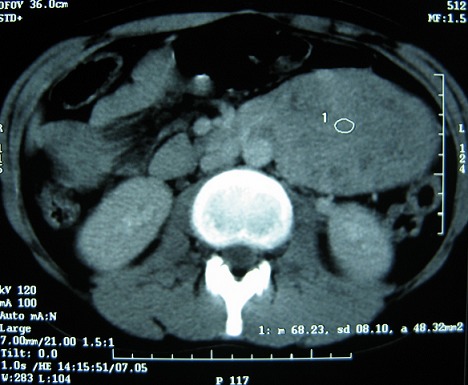
Scanner avec injection du produit de contraste montrant la prise de contraste hétérogène de la tumeur

Mlle E.F âgée de 20 ans, sans antécédents particuliers, avait consulté pour des douleurs para ombilicales gauches associées à des vomissements incoercibles post prandiaux tardifs d’évolution croissante. Il n'y avait pas de fièvre ni d'hémorragie digestive. Cette symptomatologie évoluait dans un contexte d'amaigrissement chiffré à 13 kilos en 1 mois. L'examen retrouvait une patiente amaigrie avec plis de déshydratation et de dénutrition. L'abdomen, souple, était le siège d'une masse latéro ombilicale sensible, fixe par rapport au plan profond d'environ de 10 cm de diamètre. Le reste de l'examen était sans particularité. Une échographie révélait l'existence en latéro aortique gauche d'une formation hétérogène de 8,5 x 6 cm de diamètre. Le tomodensitométrie (TDM) abdominale, réalisée avant et après injection du produit de contraste, objectivait la présence d'une tumeur occupant l'hypochondre et le flanc gauche, bien limité mesurant 9 x 7 cm, prenant le contraste et ne contenant pas de calcifications (Figure 1, Figure 2). La radiographie du thorax était sans particularité.

**Figure 3:**
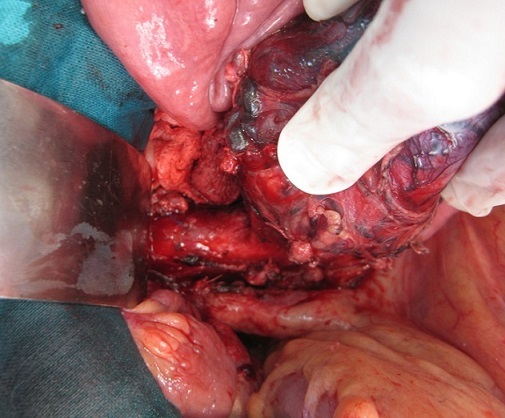
Image peropératoire après section du muscle de Treitz montrant le paragangliome retroperitoneal

**Figure 4:**
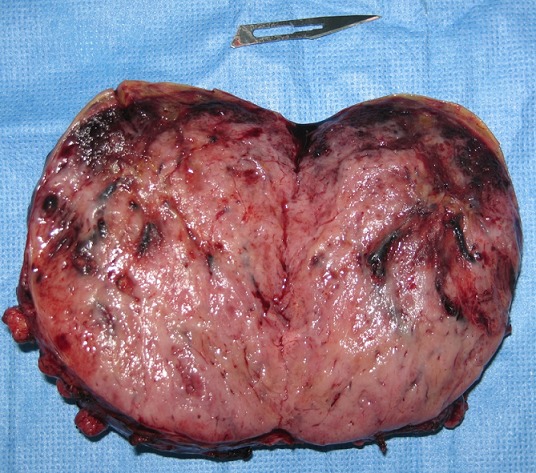
Pièce de résection ouverte

**Figure 5:**
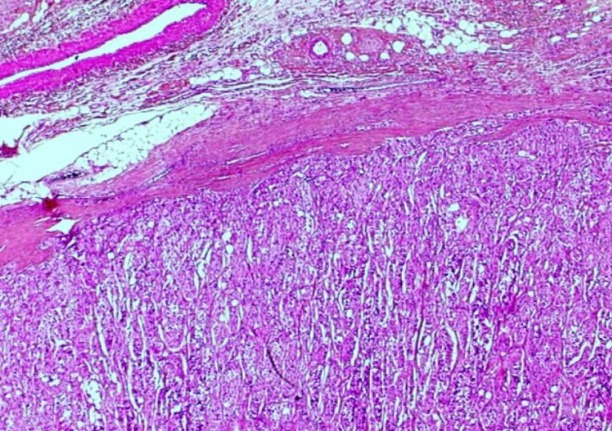
Aspect histologique du paragangliome

La fibroscopie œsogastroduodénal progressant jusqu’ au deuxième duodénum était normale. En biologie, il existait des signes de déshydratation: une insuffisance rénale fonctionnelle, une hyponatrémie, une hypokaliémie ainsi qu'une hypoprotidémie. Devant ce tableau d'occlusion haute avec retentissement hydroélectrolytique et la présence d'une masse abdominale, une intervention chirurgicale a été indiquée. L'exploration par une laparotomie médiane retrouvait une tumeur rétropéritonéale d'environ 10 cm de diamètre. Cette tumeur était ferme, rosâtre à surface hypervascularisée reposant sur la face antérolatérale gauche de l'aorte. La masse refoulait et comprimait l'angle duodéno-jéjunal (Figure 3). Une tumorectomie était réalisée après libération du tube digestif et contrôle vasculaire (il existait une branche artérielle efférente de l'aorte abdominale) (Figure 4). Les suites étaient simples. La patiente a quitté l'hôpital à j +2. L'histologie confirmait qu'il s'agissait d'un paragangliome sans signes de malignité (Figure 5). Le suivi clinique et biologique était satisfaisant. Le recul est de deux ans. Une enquête familiale n'a révélé aucune anomalie génétique particulaire.

## Discussion

Les paragangliomes sont des tumeurs rares qui se développent à partir des cellules germinales de la crête neurale, et se localisent habituellement au niveau de la médullosurrénale [1]. Moins de 10% sont extra surrénaliens. Elles peuvent être associés à d′autres pathologies rentrant dans le cadre des néoplasies endocriniennes multiples NEM 2, à une maladie de Von hippel-Lindau (VHL) et à une neurofibromatose de type 1 (maladie de Recklinghausen) [4]. En outre, elles peuvent compléter la triade de Carney associant un léiomyosarcome gastrique et un chondrome pulmonaire [5]. Les localisations ectopiques sont le plus souvent en intra abdominales touchant les chaînes sympathiques pelviennes, l'organe de Zuckerkland, la vessie, et même le rectum et le sacrum. Le paragangliome rétropéritonéal est inhabituel. Il touche le plus souvent l'adulte entre 30 et 50 ans [1, 4].

Notre observation présente deux particularités, l'occlusion haute par le syndrome tumoral et l'absence de syndrome endocrinien. Ces tumeurs sont non sécrétantes dans 40% des cas, ce qui explique à la fois l'absence de signe fonctionnel spécifique (triade de Ménard, sueurs, pas de céphalées, tachycardie, hypotension) [2]. Le plus souvent les formes rétropéritonéales non sécrétantes sont asymptomatiques et peuvent être révélées à des stades très avancés. Toutefois, des signes d'emprunt, à type de lombalgies, de pesanteur abdominale ainsi que des signes urinaires, ont été décrits [3]. Aucun cas n'a été révélé par un syndrome subocclusif haut suite à une compression de l'angle duodéno-jéjunal. En préopératoire, aucun examen ne nous a permis d'orienter le diagnostic de paragangliome. Dans cette localisation, l'imagerie pose le diagnostic de tumeur rétropéritonéale. L’échographie montre une lésion d’écho structure tissulaire, le plus souvent hétérogène contenant des zones liquidiennes, ainsi que certaines calcifications [6]. Les paragangliomes ont généralement un aspect scannographique identique à celui des phéochromocytomes surrénaliens, mais leur topographie conditionne la difficulté diagnostique [7]. Chez notre patiente, la masse prenait légèrement le contraste au scanner. L'aspect TDM le plus classique est celui d'une masse bien limitée de plus de 2 cm de diamètre (souvent entre 4 et 5 cm), massivement rehaussée par le produit de contraste [8, 9]. L'imagerie précise aussi l'extension locale de la tumeur, et permet d’évaluer son extirpabilité [7–9].

Dans notre observation, il n'y avait pas de calcifications qui pouvaient orienter le diagnostic. En plus, l′absence de signes d′hypersécrétion de catécholamines, l'indication à réaliser une scintigraphie à la méta-iodo-benzyl-guanidine (MIBG) en préopératoire est inadaptée. Pourtant, cet examen serait positif dans beaucoup de paragangliomes non fonctionnelles et trouve une place dominante dans la surveillance post opératoire ultérieure [2, 3]. L'IRM et récemment le Petscan sont très sensibles pour détecter les petites localisations infra radiologiques, les métastases de petite taille et les autres lésions ectopiques [10]. Ces examens n'ont pas été réalisés chez notre patiente, il en est de même pour les examens biologiques. Ces derniers confirment le diagnostic en cas de tumeur sécrétante [1]. Leur dosage était négatif lors de la surveillance postopératoire de notre patiente. Si l'histologie et particulièrement l'immunohistochimie confirment le diagnostic, ils ont un intérêt pronostic grâce au marquage de la protéine S [11]. Le diagnostic de malignité est très discuté, puisque seule l′apparition de métastases dans un site où il n′existe pas habituellement de tissu paraganglionnaire ou la survenue d′une récidive locale ou à distance affirmera la malignité [12].

Le traitement du paragangliome doit rentrer dans un cadre multidisciplinaire. La chirurgie complète sans résidu microscopique reste le seul traitement qui permet des taux de survies de plus de 75% à cinq ans [11]. Nous avons réalisé une laparotomie, vu la grande taille de la tumeur. Des auteurs ont rapporté la faisabilité de la coelioscopie dans les tumeurs de moins de 5 cm [13]. La radiothérapie et la chimiothérapie ont permis de contrôler dans quelques cas la maladie chez des patients métastatiques, mais le pronostic dépend de la récidive locale et des marges de résection lors de l'exérèse de la tumeur [14]. 10% des paragangliomes sont héréditaires. En effet, une mutation SDHB (succinate déshydrogénase complex subunit B) s'accompagnait d'une agressivité et d'un risque de récidive plus important que chez les patients ayant une mutation SDHD. Ces données incitent qu'une enquête génétique soit réalisée systématiquement afin de rechercher ces mutations [15].

## Conclusion

Le paragangliome rétropéritonéale est une entité rare. Le mode de révélation par un syndrome subocclusif haut secondaire à la compression de l'angle duodéno-jéjunal est exceptionnel et déroutant surtout en cas de tumeur non sécrétante. L'intérêt de l'imagerie est primordial pour le bilan préthérapeutique. Malgré les progrès de la radiologie, dans les formes rétropéritonéales non sécrétantes, le diagnostic est souvent affirmé qu'après analyse histologique de la pièce opératoire. Il faut donc savoir l’évoquer devant toute masse rétro péritonéale isolée afin d'entreprendre les précautions nécessaires pour éviter des complications qui peuvent être fatales. Le traitement doit intégrer une équipe multidisciplinaire incluant endocrinologue, anatomopathologiste et chirurgien. Seule la chirurgie curative permet de prolonger la survie. Un suivi postopératoire prolongé est recommandé afin de détecter les formes malignes.
